# Pharmacological Enhancement of Extinction Retention in Non-stressed Adolescent Rats but Not Those Exposed to Chronic Corticosterone

**DOI:** 10.3389/fnins.2022.822709

**Published:** 2022-03-16

**Authors:** Anthea A. Stylianakis, Kathryn D. Baker, Rick Richardson

**Affiliations:** School of Psychology, The University of New South Wales, Sydney, NSW, Australia

**Keywords:** adolescent, extinction, rat, tropomyosin receptor kinase B, 7, 8-dihydroxyflavone, chronic stress

## Abstract

Individuals exposed to chronic adverse experiences in childhood and adolescence are at increased risk of developing neuropsychiatric illnesses such as mood and anxiety disorders. Symptoms of anxiety disorders can often be reduced through exposure therapy, which is based on the process of extinction. Although chronic stress in adolescence is known to exacerbate the impaired extinction of learned fear during this period of development, it remains unclear whether exposure to stressors in adolescence qualitatively affects the mechanisms underlying fear extinction. Brain-derived neurotrophic factor (BDNF) and its principle receptor, tropomyosin receptor kinase B (TrkB), are involved in neuroplasticity underlying fear extinction. The small-molecule TrkB agonist 7,8-dihydroxyflavone (7,8-DHF) improves fear extinction and reduces fear relapse (reinstatement) in adult mice when administered prior to extinction training but its effects in younger ages are unknown. In this study we tested whether 7,8-DHF enhances extinction retention and leads to less renewal in both stressed and non-stressed adolescent rats. Pre-extinction injection of 7,8-DHF led to lower levels of CS-elicited freezing in both the extinction and conditioning contexts in non-stressed adolescent male rats, but not in those given 7 days of corticosterone. These findings indicate that chronic stress interferes with the effectiveness of pharmacological agonism of TrkB in enhancing fear extinction in adolescence. A greater understanding of the mechanisms underlying extinction in adolescence and the effect of chronic corticosterone exposure on those mechanisms may inform a deeper understanding of the etiology and treatment of pediatric stress-related disorders.

## Introduction

Adolescence is often termed a period of “storm and stress” (Buchanan and Hughes, 2011). Further, stress-sensitive areas of the brain, such as the prefrontal cortex, hippocampus, and amygdala, undergo substantial modification during adolescence (Teicher et al., 2016), as do several hormonal systems, including the system primarily involved in responding to acute and chronic stressors [i.e., the hypothalamic-pituitary-adrenal (HPA) axis; Romeo, 2013]. These brain regions and hormonal systems play an integral part in emotion regulation, a facet of cognition that is undergoing substantial development during adolescence (Hartley and Phelps, 2009). Hence, it is perhaps unsurprising that this period of development is one in which many psychiatric disorders, including anxiety disorders, first emerge (Beesdo et al., 2009). Furthermore, for adolescents exposed to adversity before the age of 18, the vulnerability of developing a stress-related disorder, either during adolescence or later in life, is increased (Edwards et al., 2003; Cabrera et al., 2007; Cloitre et al., 2019). It has been suggested that the link between adverse experiences in childhood and adolescence and the later development of psychiatric disorders like anxiety may be mediated by disruptions in an individual’s capacity to regulate their emotions when faced with later stressors (Burns et al., 2010; Barlow et al., 2017; Cloitre et al., 2019). Moreover, the neural and physiological systems affected by chronic stress are also those involved in emotion regulation (McEwen et al., 2015). Given that adolescence is a period of development in which the neural systems important for emotion regulation are undergoing maturation, exposure to chronic stress may have particularly profound effects on the mental health of adolescents (Tottenham and Galván, 2016). Unfortunately, many adolescents are exposed to such adversity, with Kessler et al. (2010) reporting that 40% of people are exposed to chronic stress before adulthood. While there has been increased interest in the learning and memory processes involved in emotion regulation in adolescence in the last decade (Baker and Richardson, 2017; Cisler and Herringa, 2021), the impact of chronic stress on these processes is not well understood. In other words, although adolescents are thought to be particularly vulnerable to the effects of traumatic experiences, there is little research into the impact of such experiences on processes related to emotion regulation.

One important process of emotion regulation is extinction of learned fear (Sotres-Bayon et al., 2006). A particularly robust difference in learning and memory processes reported in adolescence is diminished extinction of Pavlovian fear conditioning. Pavlovian fear conditioning refers to a behavioral paradigm where an initially neutral cue is paired with an aversive stimulus (Unconditioned Stimulus; US). This results in the cue, now referred to as a conditioned stimulus (CS), eliciting conditioned fear responses (CRs). Extinction training refers to a procedure where the CS is repeatedly presented without the US, which leads to a reduction in the CRs (Anagnostaras et al., 2015). The retention of extinction can be assessed later by presenting the CS again and involves the retrieval of a safety memory that competes for expression with the original fear memory (Bouton, 2004; Lonsdorf et al., 2019). Diminished retention of cued fear extinction is reported in adolescent rats relative to older and younger animals despite a similar reduction in fear responses during extinction training while adolescent mice exhibit deficits in extinction learning and retention of both cued and context fear (for review see Bisby et al., 2021). Diminished learning or retention of cued fear extinction has also been reported in humans (e.g., Pattwell et al., 2012; Ganella et al., 2017). As the maintenance of extinction is a challenge for exposure-based treatments for clinical anxiety and fear-related disorders in youth and adults (Rauch et al., 2012; Vervliet et al., 2013; Kodal et al., 2018), understanding the processes which strengthen extinction retention in adolescence in animal and human laboratory studies may ultimately provide insight into clinical interventions to reduce excessive fear in this age group.

Preclinical research has identified several methods which enhance extinction retention in adolescent rats, broadly falling into behavioral and pharmacological interventions. One example of a behavioral approach is doubling the amount of extinction training given to adolescents, which leads to equivalent extinction retention as observed in adult animals (e.g., McCallum et al., 2010). In terms of a pharmacological adjunct, the partial NMDA receptor agonist D-Cycloserine (DCS) improves subsequent extinction retention in adolescent rats when administered immediately following extinction training (McCallum et al., 2010), similar to its effects in adults (Walker et al., 2002; Ledgerwood et al., 2003).

An important consideration in the use of behavioral or pharmacological interventions to enhance extinction is that exposure to chronic stress can affect their efficacy in adolescent rats (Stylianakis et al., 2019). Specifically, exposure to chronic stress during adolescence impairs extinction retention even after extended extinction training. For example, in one study chronic stress during early adolescence (27–33 days old) was modeled by having rats drink corticosterone-infused water for 7 days (Den et al., 2014). This type of stressor has been shown to mimic the neural and physiological effects of other types of stress, such as repeated restraint stress and chronic social stress (Luine et al., 1993; McKittrick et al., 2000; Cook and Wellman, 2004; Radley et al., 2006; Jeong et al., 2013; Hoffman et al., 2014; Kaplowitz et al., 2016). Adolescent rats exposed to corticosterone displayed significantly higher CS-elicited freezing at the extinction retention test, as compared to rats exposed to vehicle or water, which did not differ from each other, following extended extinction training (Den et al., 2014). In another set of experiments, Stylianakis et al. (2019) replicated those effects and further reported that pharmacological enhancement of extinction retention by DCS in adolescent rats was abolished when animals had been exposed to chronic corticosterone in their drinking water. These findings suggest that two methods that have been shown to ameliorate the extinction retention deficit in non-stressed adolescent rats, extended extinction training and DCS, do not facilitate extinction retention in adolescents exposed to chronic stress. Moreover, this work provides evidence for the idea that chronic stressor exposure during adolescence has particularly deleterious effects on extinction processes (i.e., similar effects of the chronic stress were not observed in younger or older rats).

Based on these findings, alternative methods to enhance extinction retention in stress-exposed adolescents need to be explored. In addition, awareness that chronic stress can impair extinction processes could be useful in clinical settings where excessive fears are targeted with extinction (i.e., exposure; Graham and Milad, 2011). Therefore, here we examined the potential of an alternative pharmacological adjunct, 7,8-dihydroxyflavone (7,8-DHF), to improve fear extinction retention in adolescent rats exposed to chronic corticosterone. This adjunct was chosen based on a report that the administration of 7,8-DHF prior to extinction enhanced cued fear extinction in male mice (Tohyama et al., 2020), as well as a study which found that administration of 7,8-DHF prior to extinction reduced fear responses during extinction training in both non-stressed adult mice as well as those exposed to immobilization stress prior to fear conditioning (Andero et al., 2011). The non-stressed mice given 7,8-DHF also exhibited less relapse (i.e., reinstatement) of extinguished fear, compared to those given an injection of the vehicle. This adjunct is proposed to be a tropomyosin receptor kinase B (TrkB) agonist (Jang et al., 2010; Liu et al., 2014), and there is evidence 7,8-DHF upregulates phosphorylation of TrkB in the amygdala, a key region for extinction learning, when delivered systemically in mice (Andero et al., 2011). In the present study, we examined the efficacy of 7,8-DHF in facilitating fear extinction learning and retention (and reducing relapse) in non-stressed adolescent rats as well as those exposed to chronic corticosterone.

## Materials and Methods

### Subjects

Subjects were 116 experimentally naïve male Sprague-Dawley rats, bred and housed in the School of Psychology at UNSW Sydney. Rats were maintained in a humidity- and temperature-controlled room on a 12-h light/dark cycle (lights on at 0700). Animals were weaned at postnatal day (P)21-P22 and housed with two or three other rats in plastic boxes (60 cm long × 30 cm wide × 12 cm high) with wire tops (total height 27.5 cm). A maximum of one animal per litter was allocated into each experimental group. Water and food were available *ad libitum*. Animals from a given stress condition were housed together, but were randomly allocated to drug condition (i.e., 7,8-DHF or vehicle). All animals were treated in accordance with the Australian Code of Practice for the Care and Use of Animals for Scientific Purposes (8th Edition, 2013). The Animal Care and Ethics Committee at UNSW Sydney approved all procedures.

### Apparatus

All behavioral procedures occurred in two sets of chambers (24 cm long × 30 cm wide × 21 cm high; Med Associates). One set of chambers was used as Context A and the other as Context B. Each chamber was fitted with a speaker to deliver a white-noise CS. Chambers were enclosed in sound-attenuating cabinets. Each cabinet was fitted with a camera on the rear wall through which behavior was digitally recorded *via* computer-based recording software (Blue Iris). Each cabinet also contained a ventilation fan that provided a low level of background noise (∼58 dB). CS and US presentations were controlled by Med-PC V software. The chambers were cleaned with tap water after each experimental session.

#### Context A

The two identical chambers referred to as Context A were constructed of stainless-steel walls with a Perspex door and ceiling. The floor consisted of stainless-steel rods spaced 16 mm apart. Underneath the rods was a stainless-steel tray containing corncob bedding. A clear Perspex sheet divided the chamber into two triangular spaces and the rat was placed into the side that housed the speaker. The only sources of lighting in Context A were red LEDs on the ceiling of the cabinet.

#### Context B

The Context B chambers were constructed of similar materials to Context A but they differed in terms of size, visual features, lighting, and flooring. Specifically, sheets of paper with 2.5 cm vertical black-and-white stripes covered the outside of the Perspex ceiling and door in these chambers. A clear Perspex sheet covered the grid floor and there was no Perspex divider in the Context B chambers. A white light was placed on top of the chambers to provide additional lighting (∼4 lux, Deglitch light meter QM1587) to the red light.

### Procedure

#### Pellet Implantation

In experiments for Analysis 2, animals in the chronic stress condition were subcutaneously implanted with a 30 mg 7-day release corticosterone pellet (4.3 mg per day average corticosterone release; pellet was 7 mm in diameter) composed of a proprietary matrix of cholesterol, cellulose, lactose, phosphates, and stearates designed to facilitate continuous diffusion of corticosterone over 7 days (Innovative Research of America, Sarasota, FL, United States). The pellet implantation occurred 5 days before the start of the handling procedures (i.e., implantation on P28 ± 1 day) to ensure animals received 7 days of corticosterone exposure before fear conditioning. Animals housed together were implanted with pellets on the same day. Placebo pellets, purchased from the same supplier, were the same size and consisted of the same matrix without the corticosterone. Dose and duration of hormone administration were chosen based on the average daily dose consumed by rats across 7 days of corticosterone administration in drinking water in our previous studies on extinction in stressed adolescent rats (i.e., Den et al., 2014; Stylianakis et al., 2019). Before implantation of pellets, animals received a pre-emptive subcutaneous (s.c.) injection of the non-steroidal anti-inflammatory analgesic Carprofen (5 mg/kg; 1 ml/kg). Following this, rats were anaesthetized by being placed in a chamber connected to a gas nozzle delivering 1–5% isoflurane in oxygen (33 ml/min). Once the rat was anaesthetized, it was removed from the induction chamber and placed in a nosepiece that supplied the isoflurane in oxygen throughout the surgery, which did not last more than 10 min (and usually much less than that). The body temperature of the animal was maintained during and post-surgery with the use of a heat pad. Following the onset of stable anesthesia (as verified by paw pinch), an injection of 0.1 ml of the local anesthetic bupivacaine (0.5%) was given at the site of incision. Using a scalpel blade, a ∼2 cm incision was made in the skin above the scapula. The skin was pulled open using surgical skin hooks, and a corticosterone or placebo pellet was implanted 0.5 cm under the incision between the skin and muscle tissue. After the pellet had been inserted, the skin was sewn together with surgical sutures and surgical staples and Vetbond Tissue Adhesive was applied to the incision area. Post-surgical infection was minimized by injecting rats with a prophylactic dose of procaine penicillin (150 mg/ml, 0.6 ml/kg s.c.). The wellbeing of the rats was monitored daily for 7 days, which included taking their weight.

#### 7,8-DHF Administration

Rats were given an intraperitoneal (i.p.) injection of 5 mg/kg 7,8-DHF (7,8-dihydroxyflavone hydrate; Sigma-Aldrich D5446-10MG) dissolved in 17% dimethyl sulfoxide (DMSO; Sigma) and phosphate-buffered saline (PBS; pH 7.2; Andero et al., 2011) or vehicle (17% DMSO in PBS). After being dissolved in DMSO and PBS, the 7,8-DHF solution was kept refrigerated for up to 48 h. 7,8-DHF was administered 1 h prior to extinction. The injection was administered as a volume of 1 ml/kg.

#### Behavioral Procedures

The behavioral procedures started when animals were between P32 and 34, and consisted of handling and pre-exposure, fear conditioning, extinction training, an extinction-retention test, and a renewal test. Each procedure was separated by ∼24 h and occurred around the same time of day (between 15:00 and 17:00 to ensure that all animals were at a similar point in their diurnal corticosterone cycle; Maywood et al., 2007).

#### Handling and Pre-exposure

Rats were handled for 4 min each day for two consecutive days. On each of these days, all rats were pre-exposed to Context A for 8 min to familiarize them with this context.

#### Fear Conditioning

Fear conditioning occurred in Context A. Following a 2-min adaptation period, rats were given three pairings of a white noise CS (7 dB above background noise levels, 10 s duration) and a scrambled foot-shock US (0.45 mA, 1 s duration). The US was presented in the last second of the CS so that the stimuli co-terminated. The three CS-US pairings were separated by inter-trial intervals (ITIs) of 135 and 85 s (mean ITI was 110 s). These conditioning parameters were based on those used by Stylianakis et al. (2019).

#### Extinction Training

Extinction training took place in Context B to minimize the possibility that freezing at extinction could be attributed to learned fear of the context, as opposed to fear of the CS. After a 2-min adaptation period, rats received 30 non-reinforced presentations of the white noise CS (10 s each, 10 s ITI).

#### Extinction Retention Test

Extinction retention was tested in Context B. Following a 2-min adaptation period, rats received a 2-min CS presentation. The longer CS duration at test than at conditioning and extinction is a standard procedure in many of our studies on fear extinction retention in developing and adult rats (e.g., McCallum et al., 2010). However, as noted in a recent systematic review, adolescent rats exhibit comparable impairment in extinction whether the CS is presented continuously for 2 min or *via* multiple 10 s presentations (see Bisby et al., 2021).

#### Renewal Test

Renewal was tested in Context A (i.e., ABA renewal was assessed). Following a 2-min adaptation period, rats received a 2-min CS presentation.

#### Scoring

Freezing was operationalized as the absence of movement other than that necessary for respiration (Fanselow, 1980). Rats were scored as freezing or not freezing every 3 s during the adaptation (pre-CS) period as well as the CS presentations at conditioning, extinction, the extinction retention test, and the renewal test. The percentage of time spent freezing was calculated for each animal, with percentage of time freezing calculated for each of the three conditioning trials, five blocks at extinction (with each block consisting of six extinction trials), and the extinction retention and renewal tests. A random sample (∼30%) of the CS-elicited freezing at the extinction retention and renewal tests was cross-scored by an individual who was blind to the experimental condition of subjects. Inter-rater reliability was very high (*r* = 0.94–0.96 across the experiments reported here).

#### Adrenal Glands and Bodyweights

A subset of animals implanted with a corticosterone (*n* = 25, 13 injected with 7,8-DHF; included in Analysis 2) or placebo pellet (*n* = 17, 7 injected with 7,8-DHF; included in Analysis 1) were weighed on the day of extinction training and following the last behavioral test before euthanasia using carbon dioxide. The animals’ abdomens were cut laterally to expose the kidneys and the adrenal glands were excised from above the kidney with visible fat removed. The adrenal glands were weighed as a pair [on a Mettler Toledo, MTL 025-MET balance; Readability (d) = 0.1 mg].

#### Analysis

The aim of this study was to determine the efficacy of 7,8-DHF in ameliorating the extinction retention deficit in non-stressed adolescent rats and, if so, then test the efficacy of this adjunct in chronically stressed adolescent rats. Two overarching analyses were conducted, each involving pooled data from three experiments with vehicle and 7,8-DHF groups (see Supplementary Material for numbers of animals per experiment included in the analyses). Analysis 1 was conducted on data from experiments with non-stressed adolescent rats. In all three of those experiments, adolescent rats were handled for 2 days before undergoing conditioning 24 h later. The following day, the rats were injected with either 7,8-DHF or vehicle 1 h before extinction training, and 24 h after this they underwent an extinction retention test before undergoing a renewal test the following day. A subset of rats [*n* = 8 injected with 7,8-DHF (out of a total of *n* = 29 animals in the final data set), *n* = 11 injected with vehicle (out of a total of *n* = 33 in the final data set)] included in Analysis 1 were implanted with a placebo pellet at P28 (±1 day), 5 days before the first day of handling. Analysis 2 compared data collected from rats implanted with a corticosterone pellet at P28 (±1 day), which all underwent the same behavioral procedure 5 days later as in Analysis 1. The aim of Analysis 1 was to examine the efficacy of 7,8-DHF on extinction retention in non-stressed adolescent rats, while the aim of Analysis 2 was to examine the efficacy of 7,8-DHF on rats exposed to chronic corticosterone.

All statistical analyses were conducted using SPSS Version 26. A significance value of *p* = 0.05 was applied to all analyses. In all analyses, the experiment number (coded as a nominal variable) was included as a factor to detect whether any group main effects or interactions varied by the experimental replication. Pre-CS freezing data at each session was analyzed using ANOVA with group (vehicle or 7,8-DHF) and experiment as between-subjects factors. CS-elicited freezing during conditioning and extinction were analyzed using separate mixed-model ANOVAs with group and experiment as between-subjects factors and trial or block of six CSs as a repeated measure factor for conditioning and extinction analyses, respectively. When the assumption of sphericity was violated for repeated measure ANOVAs, the Greenhouse-Geisser procedure was followed to adjust degrees of freedom and *p* values. CS-elicited freezing at the extinction retention and renewal tests were analyzed using separate ANOVAs with group and experiment as between-subjects factors. Given that renewal can be viewed as the degree of relapse outside the extinction context, a subsequent mixed-model ANOVA compared freezing across groups across tests, with test as a repeated measures factor. Interactions were explored with simple main effects. Bodyweight and adrenal gland weight as a percentage of bodyweight were analyzed with 2 × 2 ANOVAs with factors of drug (vehicle or 7,8-DHF) and pellet (placebo or corticosterone). Measures of effect sizes are also given (partial η^2^ for the above analyses where small effect size = 0.001, medium effect size = 0.059, and large effect size = 0.138; Richardson, 2011).

Exclusion criteria were applied such that any rat that did not show evidence of learning the CS-US association at conditioning (<6% freezing on block 1 of extinction training) or had failed to learn during extinction (> 94% freezing across the final four blocks of extinction training) was excluded from the analysis. This resulted in the exclusion of nine rats from the 7,8-DHF group in Analysis 1, four rats from the 7,8-DHF group in Analysis 2, and one rat from the vehicle group in Analysis 2. In addition, the extinction data of three rats in the 7,8-DHF group and the renewal results of three rats in the control group of Analysis 1 were not included in the analysis due to experimenter error (e.g., recording failure). Furthermore, three of the rats that had their adrenal glands excised did not have their weight recorded at 2 days post-pellet washout due to errors in weight recording.

## Results

### Analysis 1

We initially compared the behavioral data of those implanted with a placebo pellet to those not implanted with a pellet in rats injected with 7,8-DHF or vehicle. These analyses confirmed that placebo pellet implantation did not affect behavior during any pre-CS period, conditioning, extinction, extinction retention or renewal [7,8-DHF group: largest *F*_(3.12, 74.79)_ = 2.38, *p* = 0.074, η_p_^2^= 0.090, extinction block by pellet interaction; vehicle group: largest *F*_(1,31)_ = 2.38, *p* = 0.13, η_p_^2^= 0.071, pellet effect for conditioning pre-CS]. Therefore, the subsequent analyses disregarded whether animals had pellets or not.

#### Pre-CS

Table 1 provides levels of pre-CS freezing across sessions for data included in Analysis 1. Pre-CS freezing did not differ between groups at conditioning [*F*_(1, 56)_ = 1.37, *p* = 0.25, η_p_^2^= 0.024], extinction training [*F*_(1, 53)_ = 0.27, *p* = 0.61, η_p_^2^= 0.005], the extinction retention test [*F*_(1, 56)_ = 3.39, *p* = 0.071, η_p_^2^= 0.057], or the renewal test [*F*_(1, 53)_ = 0.71, *p* = 0.79, η_p_^2^= 0.001]. An effect of experiment or interaction of experiment with group was not detected at conditioning, the extinction retention test, or the renewal test [largest *F*_(2, 53)_ = 3.00, *p* = 0.058, η_p_^2^= 0.102, experiment main effect at renewal]. Pre-CS freezing in the 7,8-DHF and vehicle groups varied at extinction training across experiments [experiment effect: *F*_(2, 53)_ = 1.24, *p* = 0.30, η_p_^2^= 0.045; interaction: *F*_(2, 53)_ = 3.57, *p* = 0.035, η_p_^2^= 0.119] such that the pre-CS freezing was slightly higher in the vehicle controls (*M* = 10.28) relative to the 7,8-DHF group (*M* = 1.25) in one out of three experiments [*F*_(1, 53)_ = 5.40, *p* = 0.024, η_p_^2^= 0.092; other *F*s ≤ 1.97, *p* ≤ 0.166, η_p_^2^≤ 0.036]. Overall, these results suggest that pre-CS freezing was relatively low across most sessions and was largely unaffected by group.

**TABLE 1:** Mean (SEM) pre-CS freezing across sessions for data included in Analysis 1.

	Vehicle *n* = 33	7,8-DHF *n* = 29
Conditioning	0.83 (0.39)	0.34 (0.20)
Extinction	3.86 (1.88)	3.08 (1.05)
Extinction retention test	7.96 (2.53)	2.41 (0.73)
Renewal test	5.33 (2.00)	6.64 (2.28)

*Due to missing cases, n = 26 at extinction training in the 7,8-DHF group and n = 30 in the vehicle group at the renewal test.*

#### Conditioning and Extinction

**FIGURE 1:**
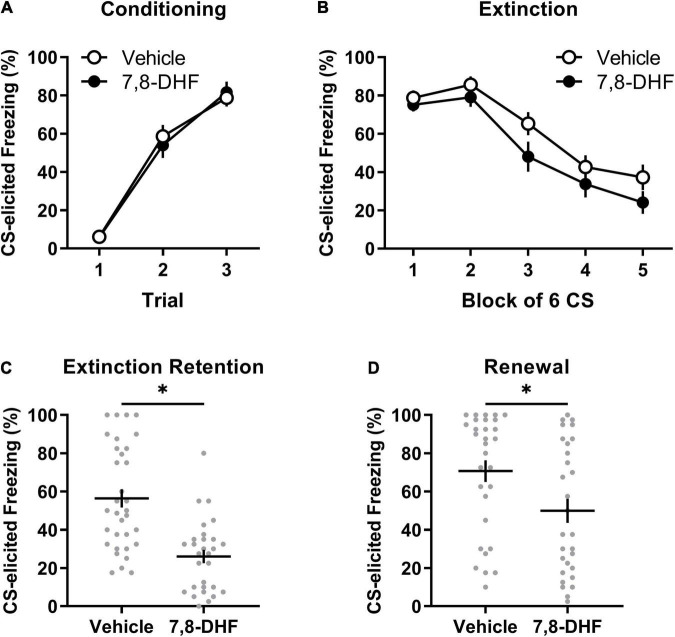
Adolescent non-stressed rats exhibit lower levels of freezing when tested in either the extinction or conditioning contexts when 7,8-DHF is combined with extinction training. Data included in Analysis 1 are represented by mean (±SEM) levels of CS-elicited freezing at Conditioning **(A)** and Extinction **(B)**. CS-elicited freezing data at the Extinction Retention **(C)** and Renewal tests **(D)** are shown as individual dot plots with mean (±SEM). Asterisk represents significant (*p* < 0.01) group difference. Group sizes were *n* = 33 for vehicle and *n* = 29 for 7,8-DHF with the exception of *n* = 26 for 7,8-DHF at extinction training and *n* = 30 for vehicle at the renewal test.

Figures 1A,B show that the 7,8-DHF and vehicle group exhibited a comparable increase in CS-elicited freezing during conditioning and a comparable decrease in CS-elicited freezing across extinction training. This description was confirmed with a mixed-model ANOVA revealing a trial main effect at conditioning [*F*_(2, 112)_ = 122.70, *p* < 0.001, η_p_^2^ = 0.687] but no group or experiment effects or interactions [largest *F*_(2, 56)_ = 1.31, *p* = 0.28, η_p_^2^ = 0.045, experiment effect].

A mixed-model ANOVA of the extinction training data detected a block main effect [*F*_(2.76, 146.28)_ = 48.34, *p* < 0.001, η_p_^2^= 0.477], but no main effects of group [*F*_(1, 53)_ = 2.48, *p* = 0.12, η_p_^2^= 0.045] or experiment [*F*_(2, 53)_ = 0.24, *p* = 0.79, η_p_^2^= 0.009], nor an interaction of block by group [*F*_(2.76, 146.28)_ = 0.52, *p* = 0.65, η_p_^2^= 0.010]. The effects of block and group did not vary by experiment [block by experiment interaction: *F*_(5.52, 146.28)_ = 1.02, *p* = 0.41, η_p_^2^= 0.037; group by experiment interaction: *F*_(2, 53)_ = 0.21, *p* = 0.82, η_p_^2^= 0.008]. Whilst a block by group by experiment interaction was detected [*F*_(5.52, 146.28)_ = 3.54, *p* = 0.003, η_p_^2^= 0.118], follow-up ANOVAs with simple main effects examining group differences across block separately across experiments did not reveal any meaningful differences in the rate of extinction between 7,8-DHF or vehicle groups; the 7,8-DHF group (*M* = 20.56) had significantly lower freezing than the vehicle group (*M* = 51.24) only at block 4 in one experiment (*p* = 0.033). Overall, these results indicate that 7,8-DHF did not affect average levels of CS-freezing or the rate of extinction.

#### Extinction Retention Test

Figure 1C illustrates that rats injected with 7,8-DHF before extinction training had lower levels of CS-elicited freezing at the extinction retention test compared to those injected with vehicle, as confirmed by a group main effect [*F*_(1, 56)_ = 25.91, *p* < 0.001, η_p_^2^= 0.316]. The group effect was consistent across experiments [largest *F*_(2, 56)_ = 0.60, *p* = 0.55, η_p_^2^= 0.021, group by experiment interaction]. This suggests that 7,8-DHF improved extinction retention in non-stressed adolescent rats.

#### Renewal

There was a significant difference in level of CS-elicited freezing between groups, with those injected with 7,8-DHF exhibiting lower levels than those injected with vehicle, suggesting less renewal in the 7,8-DHF-treated group [*F*_(1, 53)_ = 7.40, *p* = 0.009, η_p_^2^= 0.122, see Figure 1D]. The group difference was consistent across experiments [experiment effect: *F*_(2, 53)_ = 2.42, *p* = 0.10, η_p_^2^= 0.084; group by experiment interaction: *F*_(2, 53)_ = 1.42, *p* = 0.25, η_p_^2^= 0.051].

Given that renewal can be quantified as the degree of relapse when performance is tested outside of the extinction training context, a subsequent analysis examined whether each group had significant changes in freezing from the retention test (Context B) to the renewal test (Context A). A mixed-model ANOVA on CS-elicited freezing was conducted with drug (7,8-DHF or vehicle) as a between-group factor and test (extinction retention test or renewal) as a within-subjects factor. This analysis revealed a main effect of test [*F*_(1, 57)_ = 21.37, *p* < 0.001, η_p_^2^ = 0.273], indicative of renewal, and a main effect of drug [*F*_(1, 57)_ = 17.86, *p* < 0.001, η_p_^2^ = 0.239], but no significant test by drug interaction [*F*_(1, 57)_ = 2.18, *p* = 0.15, η_p_^2^ = 0.037]. These results confirm that, on average, 7,8-DHF reduced post-extinction freezing but suggest that both 7,8-DHF and vehicle groups had a comparable degree of renewal of fear outside of the extinction context.

Overall, the results of this analysis demonstrate that 7,8-DHF administered before extinction training does not affect within-session extinction but reduces fear responses at subsequent extinction retention and renewal tests in non-stressed adolescent rats. These results suggest 7,8-DHF enhances the consolidation of the extinction memory.

### Analysis 2

This analysis involved adolescent rats chronically exposed to corticosterone (*via* an implanted, slow-release pellet). Twenty-four rats were injected with 7,8-DHF and sixteen with vehicle.

#### Pre-CS

As shown in Table 2, pre-CS freezing did not differ between groups at conditioning [*F*_(1, 35)_ = 0.00, *p* = 1.00, η_p_^2^= 0.000], extinction training [*F*_(1, 35)_ = 0.79, *p* = 0.38, η_p_^2^= 0.022], extinction retention test [*F*_(1, 35)_ = 2.25, *p* = 0.14, η_p_^2^= 0.060], or renewal [*F*_(1, 35)_ = 0.61, *p* = 0.44, η_p_^2^= 0.017]. Furthermore, there were no effects of experiment or group by experiment interactions during pre-CS freezing at conditioning, extinction training, extinction retention test, or renewal [largest *F*_(1, 35)_ = 2.89, *p* = 0.10, η_p_^2^= 0.076, group by experiment interaction at extinction training].

**TABLE 2:** Mean (SEM) pre-CS freezing across sessions for data included in Analysis 2.

	Vehicle *n* = 16	7,8-DHF *n* = 24
Conditioning	1.41 (0.82)	1.04 (0.94)
Extinction	7.21 (4.62)	7.42 (2.98)
Extinction retention test	12.50 (5.76)	3.13 (1.15)
Renewal test	12.03 (5.87)	3.44 (1.80)

#### Conditioning and Extinction

**FIGURE 2:**
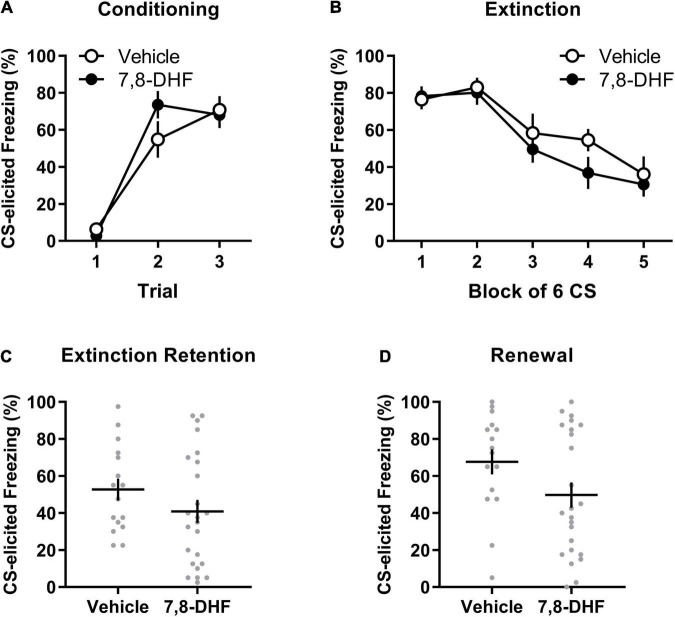
7,8-DHF combined with extinction training does not affect CS-elicited freezing during extinction training or tests of extinction retention and renewal in adolescent rats exposed to corticosterone. Data included in Analysis 2 are represented by mean (±SEM) levels of CS-elicited freezing at Conditioning **(A)** and Extinction **(B)**. CS-elicited freezing data at the Extinction Retention **(C)** and Renewal **(D)** tests are shown as individual dot plots with mean (±SEM). Group sizes were *n* = 16 for vehicle and *n* = 24 for 7,8-DHF.

Figures 2A,B show that the 7,8-DHF and vehicle groups exhibited a comparable increase in CS-elicited freezing during conditioning and a comparable decrease in CS-elicited freezing across extinction training. This description of the results was confirmed with a mixed-model ANOVA revealing a trial main effect at conditioning [*F*_(2, 70)_ = 58.16, *p* < 0.001, η_p_^2^ = 0.624] with no group or experiment effects or interactions being detected [largest *F*_(2, 35)_ = 2.46, *p* = 0.10, η_p_^2^ = 0.123, experiment effect].

A mixed-model ANOVA of the extinction data detected a block main effect [*F*_(3.03, 105.94)_ = 17.29, *p* < 0.001, η_p_^2^= 0.331] and an experiment effect [*F*_(2,35)_ = 3.47, *p* = 0.042, η_p_^2^ = 0.166]. However, Tukey’s *post hoc* tests on the experiment main effect did not detect any significant differences in average freezing across experiments (smallest *p* = 0.051). No effect of group or interactions were detected [largest *F*_(1,35)_ = 2.81, *p* = 0.10, η_p_^2^ = 0.074, group by experiment interaction].

#### Extinction Retention Test

As shown in Figure 2C, rats injected with 7,8-DHF did not exhibit significantly different levels of CS-elicited freezing compared to those injected with vehicle [*F*_(1, 35)_ = 0.001, *p* = 0.97, η_p_^2^= 0.000]. This suggests that 7,8-DHF did not improve extinction retention in chronically stressed adolescent rats. While there was no effect of experiment [*F*_(2, 35)_ = 1.53, *p* = 0.23, η_p_^2^= 0.080], there was a significant group by experiment interaction [*F*_(1, 35)_ = 4.20, *p* = 0.048, η_p_^2^ = 0.107]. This interaction was further explored by simple main effects, which found no significant effect of group within each experiment [largest *p* = 0.064, 95% CI = (−1.28, 42.95)], suggesting that group differences within experiments were not significant.

#### Renewal

The groups did not differ in level of CS-elicited freezing on the renewal test [*F*_(1, 35)_ = 1.30, *p* = 0.26, η_p_^2^= 0.036, see Figure 2D]. The group difference was consistent across experiments [experiment effect: *F*_(2, 35)_ = 1.15, *p* = 0.33, η_p_^2^= 0.062; group by experiment interaction: *F*_(1, 35)_ = 0.13, *p* = 0.72, η_p_^2^= 0.004].

As in Analysis 1, a subsequent mixed-model ANOVA examined whether each group had significant changes in CS-elicited freezing from the retention test (Context B) to the renewal test (Context A). This ANOVA had drug (7,8-DHF or vehicle) as a between-group factor and test (extinction retention test or renewal) as a within-subjects factor. This analysis revealed a main effect of test [*F*_(1, 38)_ = 8.21, *p* = 0.007, η_p_^2^ = 0.178], indicative of renewal, but no main effect of drug [*F*_(1, 38)_ = 3.01, *p* = 0.09, η_p_^2^ = 0.073] or drug by test interaction [*F*_(1, 38)_ = 0.55, *p* = 0.47, η_p_^2^ = 0.014]. These results suggest that both 7,8-DHF and vehicle groups had a comparable degree of renewal of fear outside of the extinction context.

### Adrenal Glands and Bodyweight

**FIGURE 3:**
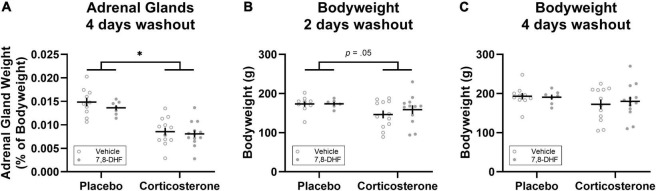
Corticosterone pellet exposure reduced adrenal gland weight as a percentage of bodyweight **(A)** and bodyweight 2 days after treatment cessation **(B)** but did not affect bodyweight 4 days after treatment cessation **(C)** relative to placebo treatment. Data is shown as individual dot plots with mean (±SEM). Asterisk represents significant (*p* < 0.001) group difference. Group sizes were *n* = 17 for the placebo (vehicle *n* = 10, 7,8-DHF *n* = 7) and *n* = 25 for the corticosterone (vehicle *n* = 12, 7,8-DHF *n* = 13) group at 4 days washout, and *n* = 15 for the placebo (vehicle *n* = 9, 7,8-DHF *n* = 6) and *n* = 24 for the corticosterone (vehicle *n* = 12, 7,8-DHF *n* = 12) group at 2 days washout.

Adrenal weights differed between groups, with the animals implanted with corticosterone pellets (*n* = 25) having smaller adrenals as a percentage of bodyweight compared to those implanted with the placebo pellets [*n* = 17; *F*_(1,38)_ = 49.23, *p* < 0.001, η_p_^2^= 0.564, as shown in Figure 3A]. Relative to those implanted with placebo pellets, animals with corticosterone pellets had lower bodyweight 2 days after corticosterone treatment cessation [*F*_(1,35)_ = 4.12, *p* = 0.050, η_p_^2^= 0.105, see Figure 3B]; however, bodyweight did not differ between groups four days after treatment cessation [*F*_(1,38)_ = 1.84, *p* = 0.18, η_p_^2^= 0.046, see Figure 3C]. There were no significant differences between adrenal gland weight and bodyweight at either 2 or 4 days washout in animals injected with 7,8-DHF or vehicle in either the corticosterone-exposed group or the group exposed to placebo [largest drug effect or interaction: *F*_(1,38)_ = 1.03, *p* = 0.32, η_p_^2^= 0.026, drug effect for adrenal glands as a percentage of bodyweight].

## Discussion

The overarching aim of the experiments reported in this paper was to firstly determine the efficacy of the TrkB agonist 7,8-DHF in ameliorating the extinction retention deficit in non-stressed adolescent rats. Upon finding that 7,8-DHF did indeed improve extinction retention in non-stressed adolescents, we sought to examine whether 7,8-DHF was efficacious in ameliorating the extinction retention deficit in chronically stressed adolescent rats. Compared to vehicle, 7,8-DHF administration (i.p.) 1 h before extinction training facilitated extinction retention (as indicated by lower levels of CS-elicited freezing) in both the extinction and the conditioning contexts in non-stressed adolescent rats (Analysis 1). However, 7,8-DHF administration had no impact in chronically stressed adolescent rats (i.e., those implanted with a 7-day-release corticosterone pellet; Analysis 2). These results contrast with those of Andero et al. (2011) and Tohyama et al. (2020) that 7,8-DHF enhanced within-session extinction in non-stressed adult mice and those exposed to immobilization stress. The possibility of a species difference is supported by the consistency of our results with reports that genetic antagonism of TrkB-mediated signaling in the amygdala of rats impairs the retention of fear extinction whilst leaving the acquisition of extinction intact (Chhatwal et al., 2006). A comparison of the results of the present study across the stressed (i.e., corticosterone-exposed) and non-stressed conditions indicates that a history of elevated stress hormone exposure interferes with the efficacy of 7,8-DHF in enhancing the maintenance of fear extinction.

Past research has shown that, compared to juvenile and adult animals, non-stressed adolescents have diminished extinction retention (McCallum et al., 2010; Pattwell et al., 2012; Bisby et al., 2021). However, this extinction retention deficit can be ameliorated by an injection of a partial NMDA receptor agonist (i.e., DCS) or giving extended extinction training (McCallum et al., 2010). In contrast, neither of these treatments facilitate extinction retention in adolescent rats exposed to 7 days of corticosterone in their drinking water (Den et al., 2014; Stylianakis et al., 2019). In the present study, 7,8-DHF was also found to facilitate extinction retention in non-stressed adolescents but not in rats exposed to chronic elevated levels of corticosterone (*via* slow release implanted pellets). Therefore, it is clear that chronic corticosterone exposure diminishes the effectiveness of at least one behavioral and two pharmacological approaches to enhancing extinction retention in adolescence.

One potential explanation for why chronic corticosterone reduces the maintenance of fear extinction in adolescent rats is that such exposure downregulates subunits of the NMDA receptors within critical brain regions that are necessary for extinction consolidation. The activation of NMDA receptors and their downstream signaling cascades (e.g., mitogen activated protein kinases) are crucial for the protein synthesis underlying the formation of long-term memories, such as extinction memories, at least in adult animals (Burgos-Robles et al., 2007; Orsini and Maren, 2012). NMDA receptors are also important for extinction retention in non-stressed adolescents, but only after extended extinction training or an injection of DCS (see Baker and Richardson, 2017). One pathway through which BDNF’s binding to TrkB receptors is hypothesized to facilitate extinction is by modulating glutamate release, resulting in increased glutamate binding to NMDA receptors, which in turn increases synaptic plasticity (Andero et al., 2011; Andero and Ressler, 2012). However, there is evidence to suggest that corticosterone exposure decreases the expression of NMDA receptor subunits in the prefrontal cortex. For example, Gourley et al. (2009) found that levels of the NMDA receptor subunit NR2B were decreased in the ventral medial prefrontal cortex (vmPFC) of adult rats that exhibited impaired extinction as a result of chronic corticosterone exposure, with the vmPFC being a region of the brain that is particularly important for extinction retention (Quirk et al., 2006). Moreover, NR2B levels in the vmPFC were correlated with extinction retention, with lower levels of NR2B being associated with poorer extinction retention. Should corticosterone exposure during adolescence also lead to a decrease in the NMDA receptor subunit NR2B, then this could be the mechanism by which the efficacy of extended extinction, DCS, and 7,8-DHF in improving extinction retention in adolescent rats is reduced (Den et al., 2014; Stylianakis et al., 2019; the present study). In order to test this hypothesis, future research could compare the phosphorylation of NMDA receptors following extended extinction, 7,8-DHF, and DCS exposure in non-stressed and chronically stressed adolescents.

As 7,8-DHF did not improve extinction retention in adolescent rats exposed to chronic stress, it is important to consider alternate means by which extinction retention can be improved in this population. In line with this, another area for future research is the examination of the efficacy of 7,8-DHF following extended extinction training in animals exposed to chronic stress. While non-stressed adolescents demonstrate good extinction retention following extended extinction training, those that have been exposed to chronic stress continue to exhibit poor extinction retention even following extended extinction training (Stylianakis et al., 2019). This suggests that adolescents exposed to chronic stress may have a weaker extinction memory relative to non-stressed adolescent rats, making it more difficult for 7,8-DHF (or DCS, as in Stylianakis et al., 2019) to enhance extinction retention. Hence, an injection of 7,8-DHF coupled with further extinction may result in a stronger extinction memory, leading to improved extinction retention.

The experiments described were not without their limitations. One limitation pertains to the use of 7,8-DHF. Whilst this adjunct was initially proposed to be a tropomyosin receptor kinase B (TrkB) agonist (Jang et al., 2010; Liu et al., 2014), and there is evidence that 7,8-DHF (at 5 mg/kg, the same dose as used in the current study) upregulates phosphorylation of TrkB in the amygdala 1 and 2 h after systemic delivery in adult mice (Andero et al., 2011), the pharmacology of 7,8-DHF is more complex than initially assumed. Several alternative targets than TrkB receptors may mediate its neurobehavioral actions *in vivo*, including activation of adenosine receptors (Pankiewicz et al., 2021). In addition, as we administered the drug systemically it is not possible to deduce whether 7,8-DHF acted centrally to facilitate extinction retention in non-stressed adolescent rats. Consequently, future experiments are needed examining the pharmacokinetics of this drug in the adolescent brain and the phosphorylation of TrkB receptors, or activation of possible alternative targets, in extinction-relevant brain regions. For example, it would be of interest to determine whether 7,8-DHF upregulates TrkB phosphorylation or neural activity in the ventral hippocampal, vmPFC, and amygdala, three regions that have been shown to be important for extinction retention, at least in adults (Chhatwal et al., 2006; Peters et al., 2010) and that are hypothesized to be under-recruited in the adolescent during the consolidation of fear extinction (Zimmermann et al., 2019). Furthermore, although a 5 mg/kg dose of 7,8-DHF was found to be effective in facilitating extinction retention in non-stressed adolescent rats in the present study, no other doses were tested. Future studies should test lower doses to establish a threshold dose (i.e., the dose at which effects are first seen) as well as higher doses (which provides information about limits and safety of higher doses), especially in chronic corticosterone-exposed adolescent rats, given that a 5 mg/kg dose of 7,8-DHF did not facilitate extinction retention in those animals.

Another limitation of the experiments reported here is that no measures of stress hormone levels in the blood of the adolescent rats were taken in order to confirm that the corticosterone pellet implantation did indeed increase circulating corticosterone levels. However, measures of adrenal glands that were taken 4 days following the cessation of corticosterone exposure show that chronic corticosterone exposure resulted in significantly reduced adrenal weights, replicating past studies with chronic exogenous corticosterone administration in the drinking water of adolescent male rats (e.g., Kaplowitz et al., 2016; Stylianakis et al., 2019). In addition, animals with corticosterone pellets had lower bodyweights 2 days after treatment cessation (i.e., on the day of extinction training) which recovered to similar levels as placebo treated animals 4 days after treatment cessation. Thus, the changes in adrenal gland weight and bodyweight confirm that administration of corticosterone *via* these slow-release pellets had a physiological effect on the adolescents in these experiments.

A third limitation of these studies derives from the way animals were exposed to chronic stress (*via* the implantation of corticosterone pellets). While exposure to chronic elevated levels of corticosterone does indeed lead to behavioral and neural changes that also occur following other chronic stress induction procedures (Luine et al., 1993; McKittrick et al., 2000; Cook and Wellman, 2004; Radley et al., 2006), an animal’s stress response consists of the release of a number of other stress hormones, each of which have specific impacts upon the brain (Charmandari et al., 2005). Therefore, it would be of interest to determine if the diminishment of 7,8-DHF’s effects on extinction following corticosterone exposure are replicated using different methods of inducing chronic stress (e.g., chronic restraint, which would result in the activation of the HPA axis in its entirety).

Future work may also seek to extend the present work in male adolescent rats by testing whether 7,8-DHF enhances fear extinction consolidation in adolescent females and whether chronic stress interferes with such an effect. Not only are fluctuations in estradiol levels across the rodent estrous cycle associated with varying effectiveness of extinction in adolescent female rats (Perry et al., 2020) but 7,8-DHF was reported to hinder extinction learning in adult female mice (Tohyama et al., 2020), or exert no influence on extinction learning, retention, or renewal (Baker-Andresen et al., 2013). Those effects in females are in stark contrast to the enhancement of fear extinction in male adult mice (Andero et al., 2011; Tohyama et al., 2020) and adolescent male rats (non-stressed) reported in the current study. Whilst age-dependent effects are possible, the possibility of sex-specific effects of 7,8-DHF on fear extinction requires addressing.

### Concluding Statement

The experiments described here demonstrate that whilst 7,8-DHF facilitates extinction retention in male non-stressed adolescents it does not facilitate extinction retention in adolescents exposed to chronic stress, at least when the same extinction conditions are used. These results add to the broader literature which has demonstrated that two other approaches that facilitate extinction retention in non-stressed rats, DCS and extended extinction, do not facilitate extinction retention in those exposed to chronic stress. These results provide further insight into the etiology and treatment of pediatric stress-related disorders, and call for further research into the mechanisms underlying the extinction retention deficit in chronically stressed adolescents, and for methods by which this deficit can be ameliorated.

## Data Availability Statement

The raw data supporting the conclusions of this article will be made available by the authors, without undue reservation.

## Ethics Statement

The animal study was reviewed and approved by Animal Care and Ethics Committee at UNSW Sydney.

## Author Contributions

AS collected all the data and wrote the first draft of the manuscript. AS and KB did all the statistical analyses. KB and RR edited the manuscript and provided funding for the study. All authors contributed to the conceptualization and design of the study, worked on subsequent drafts of the manuscript, and agreed on the final version of the manuscript.

## Conflict of Interest

The authors declare that the research was conducted in the absence of any commercial or financial relationships that could be construed as a potential conflict of interest.

## Publisher’s Note

All claims expressed in this article are solely those of the authors and do not necessarily represent those of their affiliated organizations, or those of the publisher, the editors and the reviewers. Any product that may be evaluated in this article, or claim that may be made by its manufacturer, is not guaranteed or endorsed by the publisher.
