# Supplementary material for: Pharmacological Enhancement of Extinction Retention in Non-stressed Adolescent Rats but Not Those Exposed to Chronic Corticosterone
Source: Front Neurosci. 2022 Mar 16;16:822709. doi: 10.3389/fnins.2022.822709 (PMC8966583; doi:10.3389/fnins.2022.822709)
Supplement: Supplementary file 1 [file Data_Sheet_1.PDF]

Supplementary Material

Table 1. Number of animals per experiment in Analysis 1

	Vehicle	7,8-DHF
Experiment 1	9	10
Experiment 2	11	8
Experiment 3	13	11
Total	33	29

The parameters and behavioural procedures for all experiments included in Analysis 1 were identical. The only difference between experiments was that rats in Experiment 2 were implanted with a placebo pellet.

Table 2. Number of animals per experiment in Analysis 2

	Vehicle	7,8-DHF
Experiment 1	12	12
Experiment 2	4	7
Experiment 3	-	5
Total	16	24

The parameters and behavioural procedures for all experiments included in Analysis 2 were identical. Note, Experiment 3 did not have any animals injected with vehicle as this experiment included animals injected with various doses of 7,8-DHF. Only those injected with 5 mg/kg were included in Analysis 2.
